# Prevalence, trends, and individual patterns of long-term antidepressant medication use in the adult Swiss general population

**DOI:** 10.1007/s00228-023-03559-4

**Published:** 2023-09-05

**Authors:** Melanie A. Amrein, Michael P. Hengartner, Markus Näpflin, Renato Farcher, Carola A. Huber

**Affiliations:** 1 Department of Health Sciences, Helsana Group, Zurich, Switzerland; 2 Department of Applied Psychology, Zurich University of Applied Sciences, Zurich, Switzerland; 3 Institute of Primary Care, University of Zurich, Zurich, Switzerland

**Keywords:** Antidepressants, Time trend, Pharmacoepidemiology, Long-term use

## Abstract

**Purpose:**

Antidepressant use has increased in many European countries, mostly driven by longer treatment duration. The aim of this study was to provide prevalence rates of long-term users of antidepressants for the Swiss population over the last decade and to investigate associated factors for longer use.

**Methods:**

We examined the prevalence rates of individuals with at least one prescription for antidepressants using longitudinal health claims data for 2013 to 2021. We defined short- (< one year), medium- (one–two years), and long-term users (> two years) for 2015 to 2019. We applied a binary logistic regression model to investigate the effects of population (gender, age, area of living, language, health insurance plan, and nursing home) and treatment characteristics (psychiatric or psychotherapeutic care) on long-term compared to short- and medium-term users in 2019.

**Results:**

In 2021, 9% of the Swiss population (*n* = 770,698) received at least one antidepressant prescription, which remained stable since 2013. In 2019, the proportion of long-term users was 57.4%, with steady increase since 2015. The proportion of medium- and short-term users has decreased. Older age, being a woman, living in an urban area, living in a nursing home, being enrolled in a standard care plan, and receiving psychiatric or psychotherapeutic care were factors positively associated with being a long-term user.

**Conclusion:**

The proportion of long-term users in Switzerland is high and steadily increasing. Given the ongoing debate about the confounding effects of relapse and withdrawal, more research is needed to investigate longer use of antidepressants that could indicate overprescribing.

**Supplementary Information:**

The online version contains supplementary material available at 10.1007/s00228-023-03559-4.

## Introduction

Mental health problems are a major global issue accounting for 16% of the total global burden of disease [1]. Leading causes of mental health problems are depression and anxiety disorders [2]. Antidepressants (AD) are widely used as the current standard treatment for these disorders to reduce symptoms of low mood, anxiety, or anhedonia and to prevent relapse [3, 4]. AD are also prescribed for other on- and off-label indications such as insomnia, pain, eating disorders, smoking cessation, migraine, and attention–deficit/hyperactivity disorders [5, 6]. Since their market entrance in 1980s and 1990s, global AD prescriptions increased massively especially in high-income countries [7]. In the US, the use of AD increased almost 65% between 1999 and 2014 [8]. In Europe, the mean prevalence rate over 27 countries was 7.2% in 2010, while AD consumption has doubled between 2000 and 2020 [9, 10]. This increase can be explained by several factors, for example, an increased mental health awareness or individual attitudes towards people with mental health problems [9]. However, recent literature indicates that a higher number of AD prescriptions is mainly due to a higher number of long-term users [11, 12].

The recommended duration of the use of AD depends on the underlying disorder. For example, people with major depression should use AD for at least four to nine months beyond depression remission, and people with a higher risk for recurrence should use AD for two years or more [13]. Maintenance treatments longer than two years should be clinically motivated, for example, for individuals with recurrent depression, with residual mood symptoms, or for individuals with a treatment-resistant depression [14]. Several studies showed that longer use of AD increased in the last decade [15–17]. For example, the prevalence of long-term use substantially increased by 13.7% from 1995–2005 to 2005–2015 in the Netherlands [15] and 29.9% had an AD prescription duration of at least three years between 2013 and 2016 in Italy [5]. No study so far examined prevalence of long-term use of AD in Switzerland.

Prolonged use of AD has received increasing criticism in recent years as it can increase the risk of adverse effects over time, for example, weight change, sleep disturbance, and persistent sexual dysfunction [7, 11, 18, 19]. Older people in particular have a higher risk of adverse events such as falls, stroke, or gastrointestinal bleeding [19]. It is recommended that physicians carefully assess the appropriateness of AD on an individual basis and inform the patient of any side effects, since the duration of AD use can also be influenced by physician’s and patient’s attitudes towards AD [18, 20]. To further understand the reasons for prolonged use of AD and gain insight into the factors involved, it is important to examine the characteristics of individuals with long-term use of AD.

The aim of this study was 1) to present recent one-year prevalence rates of AD prescription across the last decade in Switzerland; 2) to show prevalence trends of long-, medium-, and short-term users; and 3) to investigate relevant factors according to population and treatment characteristics associated with long-term use.

## Methods

### Data and study population

This is a retrospective cohort study based on longitudinal health claims data from the insurance group Helsana. Helsana is one of the largest health insurers in Switzerland. In 2021, it covered approximately 1,330,000 people (15%) of the Swiss population. Every person residing in Switzerland must take out health insurance. People can choose between different health insurance companies, all of which are legally obliged to cover the same healthcare costs according to the Swiss law. Every person chooses a deductible amount (franchise) between 300 and 2500 Swiss Francs. The amount indicates how much an insured person must pay themselves in a year before the health insurance company takes over the costs for health services. By choosing a higher deducible class, the insurer benefits from a premium reduction. Health insurance can either be a standard care or managed care model. For example, in the family doctor managed care model, the insured has a physician who acts as the first provider and coordinator of care when medical care is needed. Basic health insurance covers the costs in the event of illness, accident, and maternity, including health care and pharmacy invoices for health care use and prescription drugs. For the present study, we included 1) people aged 18 and older and 2) who had at least one AD prescription in the study period. To analyze prevalence rates of people with at least one AD prescription in one year, we included the years from 2013 to 2021. To analyze different categories of lengths, we compared long-, medium-, and short-term users for the years 2015 to 2019 (please see description below).

### Variables

#### Antidepressants

ADs are grouped according to the Anatomical Therapeutic Chemical (ATC) classification system of the World Health Organization with the code N06A [21]. The group is subdivided according to different modes of action: 1) tricyclic ADs (TCAs and N06AA), 2) selective serotonin reuptake inhibitors (SSRIs and N06AB), 3) monoamine oxidase inhibitors (MAOIs and N06AG), and 4) other ADs (N06AX), which have unique structures and properties that target diverse receptors in the central nervous system, e.g., serotonin norepinephrine reuptake inhibitors (SNRIs) or tetracyclic ADs (TeCA) [21]. For our analyses, we distinguished between TCAs, SSRIs, MAOIs, other ADs, and separately between mirtazapine (SNRI), venlafaxine (TeCA), and St. John’s wort (other ADs).

#### Categories of length: short-, medium-, and long-term user

To analyze the length of prescriptions, we classified people as short-, medium-, or long-term user. To do so, we tracked the first AD prescription for each user between 2013 and 2021 and excluded all people with a prescription in 2012. Without controlling for the year 2012, it would not have been possible to allocate the people to the different prescription durations, since the people could have taken AD beforehand. To define the categories of lengths, we counted the numbers of quarters with at least one AD prescription and distinguish between long-, medium-, and short-episodes. Short-term is defined as a prescription length of one or four quarters (= one year), medium-term as a prescription length of five or eight quarters (= max. two years), and long-term as a prescription length of nine or more quarters (= more than two years). If there is a break between quarters of AD prescription, we allowed for an interruption of maximum 6 months before we labeled it as a new episode of AD prescription. Examples of episodes in each category are shown in the Supplementary Fig. 1. To analyze the numbers of people by categories of length across the years, people were allocated to one of the three categories for each year. To ensure that every person has the same chance of being assigned to one of the three categories, the investigation period was set from 2015 to 2019, which is four years shorter than the total observation period. If more than one episode in one year appeared, the longest episode was counted.

#### Population characteristics

Population characteristics comprise: gender, age, age groups (19–30, 31–40, 41–50, 51–60, 61–70, 71–80, > 80), seven regions of Switzerland according to the Swiss Federal Office of Statistics (Zurich, Midland CH, Lemanic region, Northwestern Switzerland, Eastern Switzerland, Ticino, and Central Switzerland) [22], three area categories that display the level of urbanization according to the national community typology of the Swiss Federal Office of Statistics (urban, rural and intermediate area = dense urban areas and rural centres) [23], language regions (German, French, and Italian), health insurance plan (managed care vs. standard plan), franchise (low: ≤ CHF 500, high: > CHF 500), and living in a nursing home.

Treatment characteristics comprise the drug class of AD, number of prescriptions, prescription source (type of health care provider), and psychiatric or psychotherapeutic care. AD drug class includes the following categories: TCAs, SSRIs, MAOIs, mirtazapine, venlafaxine, St. John’s wort, and other ADs. Each AD prescription was coded to a prescription source that includes the following: 1) general practitioner (GP), 2) psychiatrists or psychiatric clinics, 3) hospital ambulatory and nursing homes, and 4) other medical specialists (e.g., gynaecologist). Psychiatric or psychotherapeutic care included psychiatric or psychotherapeutic diagnostics and therapies by psychiatrists or psychotherapists in psychiatric clinics or at the doctor’s office, based on the national tariff system for outpatient medical services [24].

### Statistical analysis

To analyze AD prevalence across the years, we calculated the number of people aged 18 or older who had at least one AD prescription in each year from 2013 to 2021. These numbers are provided both raw and extrapolated to the entire Swiss population using census data from the Swiss Federal Office of Statistics [25]. The procedure of extrapolation was used to adjust for age, gender, and region (of residency). The prevalence rates are calculated with the extrapolated data to give an overview of the extrapolated one-year prevalence of people with at least one AD prescription in Switzerland between 2013 and 2021.

The total number of people with at least one AD prescription between 2013 and 2021 was calculated. All results were extrapolated relative to the demographic distribution of the overall Swiss population by each year. The extrapolations were based on individual weighting factors (*wi*), which were calculated as the inverse of the sampling probability (*pi* = NHelsana,*i*/NSwitzerland,*i*) of a given stratum (*i*): *wi* = 1/*pi*. The strata are defined by people’s characteristics including age class, gender, and cantons (regions areas) by year (for more details please see Neuner-Jehle et al. [26]). The data for NSwitzerland,*i* is derived from the federal statistical office of Switzerland [25].

Chi-square tests and ANOVA were used to test differences between short-, medium-, and long-term users in population and treatment characteristics. Associations between long-term users and population and treatment characteristics were tested using logistic regression analyses. The regression analysis model included gender, age, nursing home, insurance model, psychiatric or psychotherapeutic care, living area, and language region to predict the probability to be a long-term user compared to short- and medium-term users. Results from the regression models were presented as odds ratios (ORs) with 95% confidence intervals (CI). For all tests, *p* < 0.05 was considered statistically significant. All analyses were performed using the statistical software R, version 2022.02.3 [27].

## Results

### Time trend and one-year prevalence of antidepressant use in Switzerland

In 2021, 770,698 (9.0%) individuals in Switzerland received at least one AD prescription (see Table 1). Between 2013 and 2017, there was a minor increase of 0.4% in the extrapolated one-year prevalence, but the prevalence returns to its origin in 2020. After 2020, the prevalence increased again by 0.1%. The prevalence was twice as high in women (11.6%) as in men (6.3%), and this ratio has remained stable over the years. Sample characteristics for each year, including information of sociodemographic variables, geographic indicators, AD drug classes, and health care models, are presented in the Supplementary Table 1.

**Table 1:** Extrapolated number and extrapolated one-year prevalence of people who were prescribed at least one AD in Switzerland between 2013 and 2021 in total and by gender

**Year**	**Extrapolated, *N***	**Prevalence total**	**Prevalence women**	**Prevalence men**
2013	707,291	8.9	11.5	6.1
2014	730,503	9.0	11.7	6.3
2015	734,986	9.0	11.6	6.3
2016	749,572	9.1	11.7	6.4
2017	766,559	9.2	11.8	6.6
2018	765,575	9.2	11.7	6.5
2019	762,114	9.0	11.6	6.4
2020	752,260	8.9	11.4	6.3
2021	770,698	9.0	11.6	6.3

### Long-, medium-, and short-term users: population and treatment characteristics

**Fig. 1:**
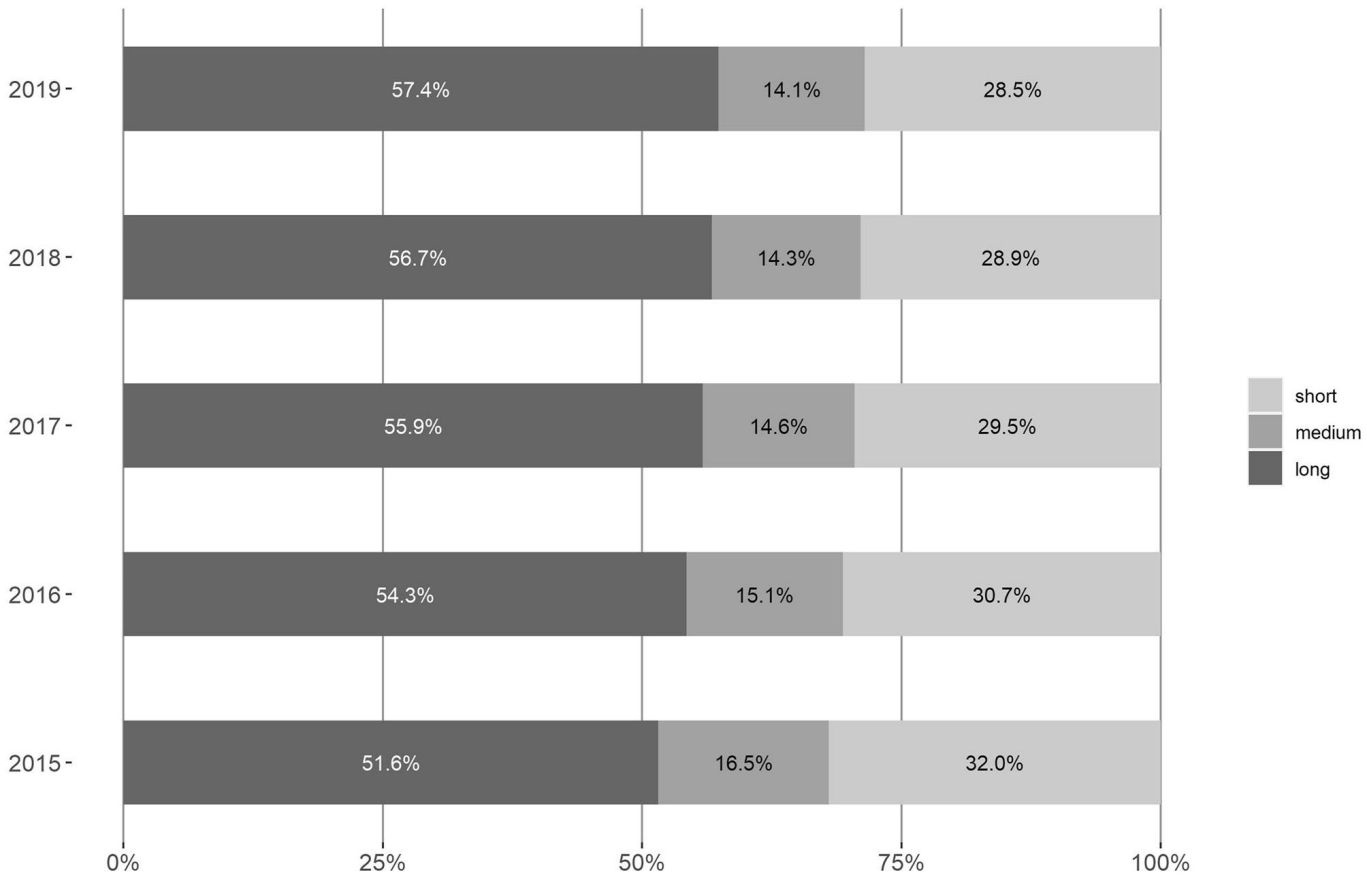
Number of people who had at least one AD prescription per year allocated to one of the three categories of length for 2015–2019

Between 2015 and 2019, the number of episodes with at least one AD prescription ranged from one to seven episodes per person, with a mean of 1.34 (SD = 0.62). Episodes were classified as long, medium, or short. From 2015 to 2019, the proportion of long-term users increased from 51.6 to 57.4%, while medium- and short-term users decreased over this period (see Fig. 1). There was a 20.6% relative increase in long-term users between 2019 (*n* = 45,988) and 2015 (*n* = 38,148). The relative decrease in medium-term users was − 7.2% and in short-term users − 3.4%. In 2019, the duration of prescription for long-term users ranged from nine quarters (> two years) to 37 quarters (> nine years) with a mean duration length of 5.5 years (SD = 2.4). 48.7% of long-term users received AD prescriptions for five years or longer and 22.6% for longer than eight years.

Table 2 shows people characteristics including descriptive, geographical, and health care variables of 2019 for short-, medium-, and long-term users (*n* = 80,144). There are significantly more women among long-term users (67.0%) than short-term (63.6%) and medium-term users (63.7%), *Χ*^2^(2, *N* = 80,144) = 98, *p* < 0.001. The total mean age was 60.4 years, while long-term users were significantly older than the other groups. The distribution of short-, medium-, and long-term users varies across age groups. 22.0% of the long-term users compared to 13.3% of short-term users were older than 80 years. 56.1% of long-term users were older than 60 years. Further, Table 2 shows that more long-term users were living in a nursing home and in the Italian part of Switzerland than short- and medium-term users. More people with a standard care insurance model (vs. managed care model) were long-term users than short- and medium-term users and long-term users seemed to choose more often a low franchise. There is a small effect between the categories of lengths in terms of area of living, as long-term users have a slightly higher percentage of people living in rural areas and smaller percentage of people living in urban areas.

**Table 2:** Population and treatment characteristics: descriptive, geographical, and health care variables, AD drug classes, prescription source, and psychiatric or psychotherapeutic care by categories of length of AD prescription including difference statistics for 2019

	Total	Short	Medium	Long	
	Mean (SD)/*n* (%)	Mean (SD)/*n* (%)	Mean (SD)/*n* (%)	Mean (SD)/*n* (%)	*p*
***N***	80,144	22,858 (28.5%)	11,298 (14.1%)	45,988 (57.4%)	
**Gender (women)**	52,531 (65.5%)	14,530 (63.6%)	7200 (63.7%)	30,801 (67.0%)	< 0.001^a^
**Age**	60.4 (19.4)	55.5 (19.8)	57.6 (20.3)	63.5 (18.4)	< 0.001^b^
**Age group**					< 0.001^a^
19–30	6295 (7.9%)	2908 (12.7%)	1204 (10.7%)	2183 (4.7%)	
31–40	7970 (9.9%)	3027 (13.2%)	1462 (12.9%)	3481 (7.6%)	
41–50	11,513 (14.4%)	3674 (16.1%)	1739 (15.4%)	6100 (13.3%)	
51–60	14,606 (18.2%)	4168 (18.2%)	2026 (17.9%)	8412 (18.3%)	
61–70	11,688 (14.6%)	2907 (12.7%)	1404 (12.4%)	7377 (16.0%)	
71–80	12,926 (16.1%)	3141 (13.7%)	1479 (13.1%)	8306 (18.1%)	
> 80	15,146 (18.9%)	3033 (13.3%)	1984 (17.6%)	10,129 (22.0%)	
**Region of residence**					< 0.001^a^
Zurich	19,049 (23.8%)	5759 (25.2%)	2747 (24.3%)	10,543 (22.9%)	
Midland	16,654 (20.8%)	4515 (19.8%)	2287 (20.2%)	9852 (21.4%)	
Lemanic region	12,610 (15.7%)	3556 (15.6%)	1831 (16.2%)	7223 (15.7%)	
Northwestern	10,844 (13.5%)	3088 (13.5%)	1500 (13.3%)	6256 (13.6%)	
Eastern	9212 (11.5%)	2654 (11.6%)	1267 (11.2%)	5291 (11.5%)	
Ticino	6376 (8.0%)	1610 (7.0%)	880 (7.8%)	3886 (8.5%)	
Central	5399 (6.7%)	1676 (7.3%)	786 (7.0%)	2937 (6.4%)	
**Language region**					< 0.001^a^
German	58,375 (72.8%)	16,896 (73.9%)	8146 (72.1%)	33,333 (72.5%)	
French	15,194 (19.0%)	4293 (18.8%)	2240 (19.8%)	8661 (18.8%)	
Italian	6575 (8.2%)	1669 (7.3%)	912 (8.1%)	3994 (8.7%)	
**Area**					= 0.003^a^
Urban	55,223 (68.9%)	15,982 (69.9%)	7850 (69.5%)	31,391 (68.3%)	
intmed	15,076 (18.8%)	4264 (18.7%)	2085 (18.5%)	8727 (19.0%)	
Rural	9845 (12.3%)	2612 (11.4%)	1363 (12.1%)	5870 (12.8%)	
**Standard care model**	37,964 (47.4%)	8940 (39.1%)	4764 (42.2%)	24,260 (52.8%)	< 0.001^a^
**Franchise (low)**	68,538 (85.5%)	18,579 (81.3%)	9307 (82.4%)	40,652 (88.4%)	< 0.001^a^
**Nurs (yes)**	6852 (8.5%)	834 (3.6%)	1031 (9.1%)	4987 (10.8%)	< 0.001^a^
**AD drug class**					
TCA	7938 (9.9%)	2250 (9.8%)	1091 (9.7%)	4597 (10.0%)	= 0.500
SSRI	33,126 (41.3%)	5235 (22.9%)	4323 (38.3%)	23 568 (51.2%)	< 0.001^a^
MAOI	98 (0.1%)	10 (0.0%)	14 (0.1%)	74 (0.2%)	< 0.001^a^
Mirtazapine	10,722 (13.4%)	2456 (10.7%)	1470 (13.0%)	6796 (14.8%)	< 0.001^a^
Venlafaxine	5450 (6.8%)	562 (2.5%)	504 (4.5%)	4384 (9.5%)	< 0.001^a^
St. John’s wort	5930 (7.4%)	2653 (11.6%)	1105 (9.8%)	2172 (4.7%)	< 0.001^a^
Other ADs	21,127 (26.4%)	4682 (20.5%)	2959 (26.2%)	13,486 (29.3%)	< 0.001^a^
**Number of prescriptions**	3.1 (2.9)	1.1 (1.1)	2.5 (2.3)	4.3 (3.0)	< 0.001^a^
**Prescription source**					
GP	48,404 (60.4%)	11,293 (49.4%)	6239 (55.2%)	30,872 (67.1%)	< 0.001^a^
Psychiatrists/psychiatric clinics	18,071 (22.5%)	2893 (12.7%)	2557 (22.6%)	12,621 (27.4%)	< 0.001^a^
Hospital	8647 (10.8%)	1571 (6.9%)	1251 (11.1%)	5825 (12.7%)	< 0.001^a^
Other prescription source	6658 (8.3%)	1589 (7.0%)	880 (7.8%)	4189 (9.1%)	< 0.001^a^
**Psychiatric or psychotherapeutic care**	27,994 (34.9%)	6002 (26.3%)	4323 (38.3%)	17,669 (38.4%)	< 0.001^a^

Area: intermediate area (intmed): dense urban areas and rural centers; Franchise: high ≥ 500 CHF, low ≤ 500 CHF

*CH* Switzerland, *intmed* intermediate area, *Nurs* nursing home, *TCA* tricyclic antidepressants (N06AA), *SSRI* selective serotonin reuptake inhibitors (N06AB), *MAOI* monoamine oxidase inhibitors (N06AG), *GP* general practitioner, *others* combination of providers

^a^Pearson's chi-square test

^b^ANOVA

Treatment characteristics for drug classes, prescription sources, and psychiatric or psychotherapeutic care are also presented in Table 2. The AD class SSRI was most often prescribed across all AD classes and significantly more often for long-term users (51.2%) than for short- (22.9%) and medium-term users (38.3%). AD class MAOI, mirtazapine, and Venlafaxine were also more prescribed to long-term users, and St. John’s wort were more prescribed to short-term users. No difference between categories was found for tricyclic AD drugs. In total, general practitioners were more frequently prescribing AD (67.1%) compared to psychiatrists or psychiatric clinics (27.4%), hospitals (12.7%), and other prescription source (9.1%). In total, only 34.96% (*n* = 27,994) received psychiatric or psychotherapeutic care with the highest percentage for long-term users (38.4%) compared to medium-term (38.3%) and short-term users (26.3%).

Table 3 shows results from a logistic regression predicting the probability to be a long-term user compared to short- and medium-term user. Factors related to long-term users were higher age (OR = 1.026), being female (OR = 1.116), living in a nursing home (OR = 1.206), standard care insurance (OR = 1.451), and receiving psychiatric or psychotherapeutic care (OR = 2.292). People living in rural areas (OR = 1.164) had a higher probability to be a long-term user compared to people living in urban areas. There was no significant effect for the variable language.

**Table 3:** Binary logistic regression model predicting long-term user as opposed to short- and medium-term user for 2019

		OR	95% CI	*p*-value
Gender (women)		1.116	1.082, 1.151	< 0.001
Age		1.026	1.025, 1.027	< 0.001
Nurs		1.206	1.135, 1.281	< 0.001
Standard care (vs. managed care)		1.451	1.409, 1.495	< 0.001
Psychiatric or psychotherapeutic care		2.292	2.215, 2.372	< 0.001
Area				
Urban		1		
intmed	1.074		1.034, 1.115	< 0.001
Rural	1.164		1.113, 1.218	< 0.001
Language				
Italian		1		
German	0.972		0.920, 1.026	0.305
French	0.989		0.930, 1.052	0.733

*Nurs* nursing home, *intmed* intermediate area: dense urban areas and rural centers

## Discussion

There were three main findings in this study. First, AD prevalence in Switzerland has remained stable from 2013 to 2021. Prevalence rates for women were twice as high than for men, which did not change over the years. Second, the prevalence of long-term users compared to medium- and short-term users were higher and has been increasing from 2015 to 2019. In 2019, more than 50% of our sample were long-term users, and one in five long-term users received AD for more than eight years. Third, long-term users had a higher probability to be older, female, living in a nursing home, living in a rural area, and receiving psychiatric or psychotherapeutic care.

The extrapolated one-year prevalence in Switzerland for people with at least one AD prescription per year was 9.0% (*n* = 770 000) in 2021. Between 2013 and 2021, the one-year prevalence remained stable between 8.9–9.2%. This may surprise as for other industrialized countries prevalence rates significantly increased in the last two decades, although these rates varies widely across countries and subgroups [8, 10]. For Switzerland, AD consumption has increased until 2014 [28], but did not increase between 2017 and 2020 [29]. This is in line with our results indicating a stable prevalence after 2013. One explanation for this result could be that the prevalence rates of AD use reached the prevalence of mental disorders treated with AD. There is only little evidence on the prevalence rates of mental disorders in Switzerland. For example, in 2017, 5.4% of the Swiss population have indicated in interviews that they had a diagnosed depression, while 34.6% reported mild to severe depressive symptoms [29]. However, guidelines recommend that AD should not generally be used as first-line treatment for mild depression, because there is no statistically demonstrable difference between placebo and AD [13]. Besides depressive disorders, ADs are prescribed for other reasons such as anxiety disorder [6] and other on- and off-label indications such as eating disorders or attention–deficit/hyperactivity disorders [6]. In Switzerland, there are no studies on the current prevalence rates of these diseases. Studies on the prevalence rates are strongly needed, as they are crucial for identifying patterns of overprescribing in people with certain mental disorders.

This study shows that long-term use compared to short- and medium-term use is highly substantial and, in line with previous findings, long-term use has steadily increased between 2015 and 2019 [15, 17, 30, 31]. In our study, the prescription duration of long-term users ranged from two to nine years. Then, 48.7% of long-term users have a prescription duration of five years or more, suggesting possible chronic use [17]. The trend of an increasing number of long-term users can have different reasons. For example, there is evidence that general practitioners continue to prescribe AD because they fear relapsing symptoms of depression and because they are unaware of the negative aspects of long-term use of AD [32]. Our study shows that only a third of long-term users receive psychotherapeutic or psychiatric care, which is important to support people who cannot discontinue antidepressant therapy for fear of relapse [20]. This would lead to overprescribing of long-term AD treatment. In addition, a recent review article shows that withdrawal symptoms, such as increased anxiety, dizziness, mood swings, flu-like symptoms, insomnia, or nausea, occurs in more than half of all AD users [33] and that guidelines on AD withdrawal need to be urgently updated [34]. There is growing evidence that depression relapse is seriously confounded with withdrawal reactions, especially when doctors are not familiar with antidepressant withdrawal [35, 36]. As thousands of individuals are seeking support for AD withdrawal [37], more education, guidance, and support for general practitioners and patients are needed to reduce unnecessary treatment [11]. Another explanation for the increasing number of long-term users can be that AD are more prescribed for reasons other than depression or anxiety disorders, which may require a different treatment duration of AD, for example, chronic pain [38]. There is evidence that chronic pain was the most common potential treatment indication for newer use of AD in older adults [39]. In summary, as long-term use of AD increases, further research is needed examining different strategies that may reduce prolonged treatment.

In line with other studies, our results indicate that older people are more often long-term users [17]. Then, 56.1% of long-term users were older than 60 years. Longer use of AD in older people is problematic as these medications are often prescribed for off-label indications such as sleep disorders, without much supporting scientific evidence [40]. In addition, older people have a greater risk for adverse events, because multimorbidity and polypharmacy can lead to drug–drug interactions [19]. Psychotherapy should be considered as alternative treatment especially for long-term users, as recent meta-analyses indicate that psychotherapy is superior in longer treatment than AD treatment alone [41, 42]. Although a higher percentage of long-term users receiving psychiatric or psychotherapeutic care compared to medium- and short-term users, only 34.9% of all people with AD prescriptions also receive psychiatric and psychotherapeutic care. Further investigations are urgently needed to assess the underlying reasons.

This study has also several limitations. Firstly, our data reflect medication acquisition rather than actual consumption. However, the data are based on objective, claim-based data, avoiding reporting and recall biases that can occur when people self-report their AD use. Secondly, we included recent claims that were submitted until mid of 2021, but receipts may be submitted later, which would lead to an underestimation of long-term AD user. Thirdly, to compare percentages of long-, medium-, and short-term users across the years, we excluded all people with an AD prescription in 2013. This may have underestimated long-term users across all years, as people with prescription start in 2013 and a duration longer than nine years do not appear. Fourthly, people in Switzerland can change their health insurance every year. For this reason, we only included people with ongoing insurance coverage in the study period.

In conclusion, the one-year prevalence of people receiving at least one AD prescription in Switzerland remained stable from 2013 to 2021. Another key finding is that more than 50% of the individuals had been prescribed AD in 2019 for more than two years, with a steady increase since 2015. Interestingly, our study shows that only a third of long-term users receive psychotherapeutic or psychiatric care, which is important to support people who cannot discontinue antidepressant therapy for fear of relapse. Physicians should carefully consider the appropriateness of AD on an individual basis and inform about the increased risks of long-term use in terms of withdrawal symptoms and adverse events to avoid overprescribing long-term antidepressant use.

### Supplementary Information

Below is the link to the electronic supplementary material.

Supplementary file1 (DOCX 86 KB)

Supplementary file2 (DOCX 55 KB)

## Data Availability

Due to Swiss law restrictions, data are not publicly available. Insurance claims data from Helsana Group underlie protection and privacy restrictions, but are available upon reasonable request from Helsana Department of Health Sciences (C.A.Huber).

## Code availability

This is not applicable.
