# Supplementary figures and images for: Prevalence, trends, and individual patterns of long-term antidepressant medication use in the adult Swiss general population
Source: Eur J Clin Pharmacol. 2023 Sep 5;79(11):1505–13. doi: 10.1007/s00228-023-03559-4 (PMC10618304; doi:10.1007/s00228-023-03559-4)

**Supplementary Figure 1**. Definition of short-, medium-, and long-term users with examples.


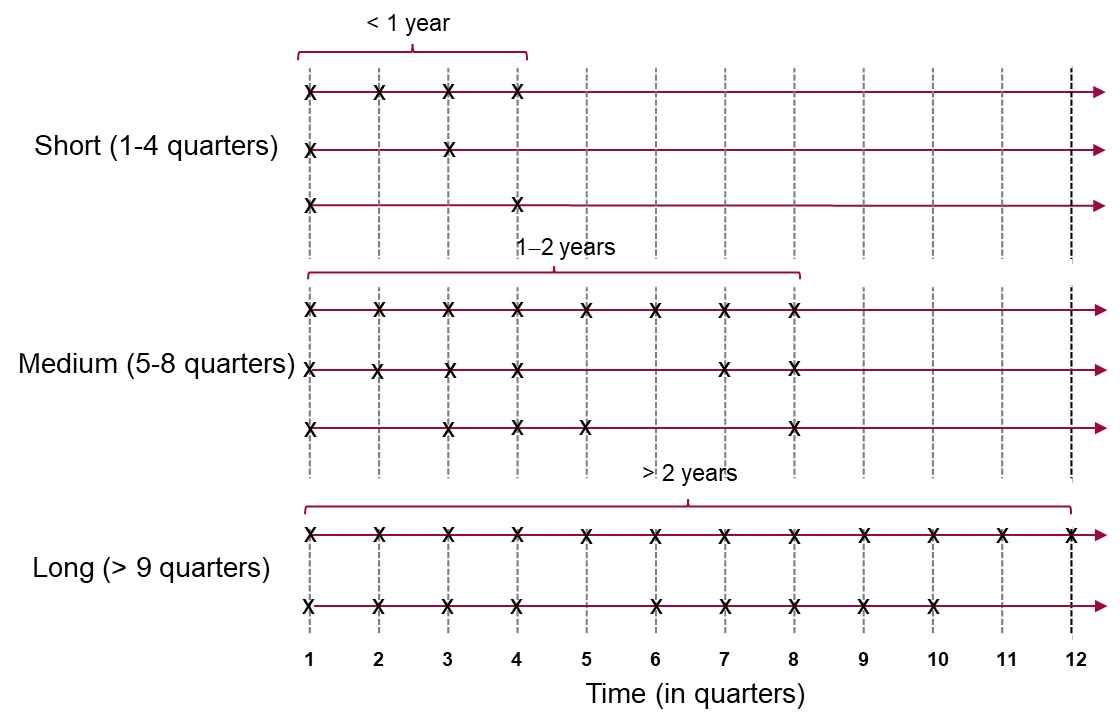

Supplement: Supplementary file 1 — Supplementary file1 (DOCX 86 KB) [file 228_2023_3559_MOESM1_ESM.docx]
