# Supplementary material for: Prevalence, trends, and individual patterns of long-term antidepressant medication use in the adult Swiss general population
Source: Eur J Clin Pharmacol. 2023 Sep 5;79(11):1505–13. doi: 10.1007/s00228-023-03559-4 (PMC10618304; doi:10.1007/s00228-023-03559-4)
Supplement: Supplementary file 2 — Supplementary file2 (DOCX 55 KB) [file 228_2023_3559_MOESM2_ESM.docx]

**Supplementary Table 1**. Characteristics of study population for 2013-2021. Each year represents the number of people, who had at least one prescription in the according year. Multiple counts possible.

|  | #Total | 2013 | 2014 | | 2015 | 2016 | | 2017 | | 2018 | | 2019 | | 2020 | | 2021 |
| --- | --- | --- | --- | --- | --- | --- | --- | --- | --- | --- | --- | --- | --- | --- | --- | --- |
|  | *n* (%) or *M* / *SD* | *n* (%) or *M* / *SD* | | *n* (%) or *M* / *SD* | *n* (%) or *M* / *SD* | | *n* (%) or *M* / *SD* | | *n* (%) or *M* / *SD* | | *n* (%) or *M* / *SD* | | *n* (%) or *M* / *SD* | | *n* (%) or *M* / *SD* | *n* (%) or *M* / *SD* |
| **N** | 970'912 | 106'087 (10.9%) | 106'814 (11.0%) | | 106'611 (11.0%) | 105'627 (10.9%) | | 102'442 (10.6%) | | 104'191 (10.7%) | | 107'231 (11.0%) | | 112'782 (11.6%) | | 119'127 (12.3%) |
| **Sex (women)** | 649'083 (66.9%) | 71'447 (67.3%) | 71'932 (67.3%) | | 71'497 (67.1%) | 70'697 (66.9%) | | 68'410 (66.8%) | | 69'519 (66.7%) | | 71'488 (66.7%) | | 74'933 (66.4%) | | 79'160 (66.5%) |
| **Age** | 59.8 / 18.911 | 59.2 / 18.497 | 59.4 / 18.604 | | 59.7 / 18.643 | 59.9 / 18.698 | | 60.4 / 18.775 | | 60.4 / 18.983 | | 60.4 / 19.207 | | 59.9 / 19.244 | | 59.0 / 19.379 |
| **Age category** |  |  |  | |  |  | |  | |  | |  | |  | |  |
| 19-30 | 69'658 (7.2%) | 7'008 (6.6%) | 7'199 (6.7%) | | 7'049 (6.6%) | 7'115 (6.7%) | | 6'878 (6.7%) | | 7'527 (7.2%) | | 7'996 (7.5%) | | 8'705 (7.7%) | | 10'181 (8.5%) |
| 31-40 | 100'516 (10.4%) | 11'811 (11.1%) | 11'665 (10.9%) | | 11'289 (10.6%) | 10'809 (10.2%) | | 9'974 (9.7%) | | 9'973 (9.6%) | | 10'383 (9.7%) | | 11'608 (10.3%) | | 13'004 (10.9%) |
| 41-50 | 149'050 (15.4%) | 17'658 (16.6%) | 17'058 (16.0%) | | 16'824 (15.8%) | 16'289 (15.4%) | | 15'335 (15.0%) | | 15'205 (14.6%) | | 15'540 (14.5%) | | 16'885 (15.0%) | | 18'256 (15.3%) |
| 51-60 | 182'700 (18.8%) | 19'694 (18.6%) | 20'012 (18.7%) | | 20'075 (18.8%) | 20'133 (19.1%) | | 19'430 (19.0%) | | 19'663 (18.9%) | | 20'131 (18.8%) | | 21'161 (18.8%) | | 22'401 (18.8%) |
| 61-70 | 152'060 (15.7%) | 17'482 (16.5%) | 17'391 (16.3%) | | 17'208 (16.1%) | 16'866 (16.0%) | | 16'197 (15.8%) | | 16'274 (15.6%) | | 16'318 (15.2%) | | 16'898 (15.0%) | | 17'426 (14.6%) |
| 71-80 | 151'934 (15.6%) | 15'965 (15.0%) | 16'383 (15.3%) | | 16'560 (15.5%) | 16'634 (15.7%) | | 16'656 (16.3%) | | 17'006 (16.3%) | | 17'161 (16.0%) | | 17'586 (15.6%) | | 17'983 (15.1%) |
| >80 | 164'994 (17.0%) | 16'469 (15.5%) | 17'106 (16.0%) | | 17'606 (16.5%) | 17'781 (16.8%) | | 17'972 (17.5%) | | 18'543 (17.8%) | | 19'702 (18.4%) | | 19'939 (17.7%) | | 19'876 (16.7%) |
| **Region** |  |  |  | |  |  | |  | |  | |  | |  | |  |
| Zurich | 220'308 (22.7%) | 24'332 (22.9%) | 24'649 (23.1%) | | 24'427 (22.9%) | 24'584 (23.3%) | | 24'115 (23.5%) | | 24'103 (23.1%) | | 24'431 (22.8%) | | 24'573 (21.8%) | | 25'094 (21.1%) |
| Midland CH | 202'808 (20.9%) | 21'396 (20.2%) | 21'550 (20.2%) | | 21'557 (20.2%) | 21'278 (20.1%) | | 20'991 (20.5%) | | 22'194 (21.3%) | | 22'856 (21.3%) | | 24'409 (21.6%) | | 26'577 (22.3%) |
| Lemanic region | 158'680 (16.3%) | 16'306 (15.4%) | 16'660 (15.6%) | | 17'237 (16.2%) | 17'015 (16.1%) | | 16'364 (16.0%) | | 16'549 (15.9%) | | 17'348 (16.2%) | | 19'635 (17.4%) | | 21'566 (18.1%) |
| Northwestern CH | 128'881 (13.3%) | 14'079 (13.3%) | 14'033 (13.1%) | | 14'013 (13.1%) | 13'962 (13.2%) | | 13'657 (13.3%) | | 14'064 (13.5%) | | 14'391 (13.4%) | | 15'005 (13.3%) | | 15'677 (13.2%) |
| Eastern CH | 118'817 (12.2%) | 14'113 (13.3%) | 14'093 (13.2%) | | 13'816 (13.0%) | 13'472 (12.8%) | | 12'620 (12.3%) | | 12'439 (11.9%) | | 12'405 (11.6%) | | 12'731 (11.3%) | | 13'128 (11.0%) |
| Ticino | 74'181 (7.6%) | 7'999 (7.5%) | 8'022 (7.5%) | | 7'928 (7.4%) | 7'830 (7.4%) | | 7'578 (7.4%) | | 7'798 (7.5%) | | 8'771 (8.2%) | | 8'999 (8.0%) | | 9'256 (7.8%) |
| Central CH | 67'237 (6.9%) | 7'862 (7.4%) | 7'807 (7.3%) | | 7'633 (7.2%) | 7'486 (7.1%) | | 7'117 (6.9%) | | 7'044 (6.8%) | | 7'029 (6.6%) | | 7'430 (6.6%) | | 7'829 (6.6%) |
| **Language region** |  |  |  | |  |  | |  | |  | |  | |  | |  |
| German | 698'899 (72.1%) | 78'003 (73.7%) | 78'262 (73.4%) | | 77'563 (72.9%) | 77'081 (73.1%) | | 74'857 (73.2%) | | 75'538 (72.6%) | | 76'455 (71.4%) | | 78'958 (70.1%) | | 82'182 (69.1%) |
| French | 193'471 (20.0%) | 19'593 (18.5%) | 20'015 (18.8%) | | 20'602 (19.4%) | 20'235 (19.2%) | | 19'533 (19.1%) | | 20'394 (19.6%) | | 21'531 (20.1%) | | 24'361 (21.6%) | | 27'207 (22.9%) |
| Italian | 76'599 (7.9%) | 8'284 (7.8%) | 8'299 (7.8%) | | 8'188 (7.7%) | 8'091 (7.7%) | | 7'851 (7.7%) | | 8'056 (7.7%) | | 9'040 (8.4%) | | 9'267 (8.2%) | | 9'523 (8.0%) |

| **Area** |  |  |  |  |  |  |  |  |  |  |
| --- | --- | --- | --- | --- | --- | --- | --- | --- | --- | --- |
| urban | 670'377 (69.0%) | 73'752 (69.5%) | 74'042 (69.3%) | 73'972 (69.4%) | 73'076 (69.2%) | 70'664 (69.0%) | 71'913 (69.0%) | 74'045 (69.1%) | 77'440 (68.7%) | 81'473 (68.4%) |
| intmed | 182'841 (18.8%) | 19'536 (18.4%) | 19'773 (18.5%) | 19'807 (18.6%) | 19'811 (18.8%) | 19'404 (18.9%) | 19'687 (18.9%) | 20'282 (18.9%) | 21'628 (19.2%) | 22'913 (19.2%) |
| rural | 117'694 (12.1%) | 12'799 (12.1%) | 12'999 (12.2%) | 12'832 (12.0%) | 12'740 (12.1%) | 12'374 (12.1%) | 12'591 (12.1%) | 12'904 (12.0%) | 13'714 (12.2%) | 14'741 (12.4%) |
| **Franchise (low)** | 867'734 (89.4%) | 95'911 (90.4%) | 96'612 (90.4%) | 96'347 (90.4%) | 95'266 (90.2%) | 92'485 (90.3%) | 93'404 (89.6%) | 95'401 (89.0%) | 99'085 (87.9%) | 103'223 (86.6%) |
| **Managed care (standard)** | 449'660 (46.3%) | 63'050 (59.4%) | 59'703 (55.9%) | 54'879 (51.5%) | 51'660 (48.9%) | 47'830 (46.7%) | 45'001 (43.2%) | 43'589 (40.6%) | 42'349 (37.5%) | 41'599 (34.9%) |
| **Nurs (Yes)** | 106'150 (10.9%) | 10'665 (10.1%) | 10'962 (10.3%) | 11'419 (10.7%) | 11'220 (10.6%) | 11'230 (11.0%) | 11'862 (11.4%) | 13'107 (12.2%) | 12'894 (11.4%) | 12'791 (10.7%) |
| **No. of prescriptions** | 3.7 / 2.699 | 3.5 / 2.562 | 3.5 / 2.602 | 3.6 / 2.611 | 3.6 / 2.658 | 3.7 / 2.701 | 3.7 / 2.716 | 3.7 / 2.781 | 3.8 / 2.802 | 3.8 / 2.810 |
| **ATC** |  |  |  |  |  |  |  |  |  |  |
| TCA | 117'776 (12.1%) | 15'817 (14.9%) | 14'890 (13.9%) | 13'847 (13.0%) | 13'222 (12.5%) | 11'958 (11.7%) | 11'785 (11.3%) | 11'960 (11.2%) | 12'079 (10.7%) | 12'218 (10.3%) |
| SSRI | 488'703 (50.3%) | 54'886 (51.7%) | 55'554 (52.0%) | 55'095 (51.7%) | 54'167 (51.3%) | 51'570 (50.3%) | 51'974 (49.9%) | 52'941 (49.4%) | 54'945 (48.7%) | 57'571 (48.3%) |
| MAOI | 2'036 (0.2%) | 298 (0.3%) | 268 (0.3%) | 256 (0.2%) | 254 (0.2%) | 213 (0.2%) | 208 (0.2%) | 186 (0.2%) | 169 (0.1%) | 184 (0.2%) |
| Others | 524'960 (54.1%) | 53'833 (50.7%) | 55'074 (51.6%) | 55'723 (52.3%) | 56'284 (53.3%) | 55'917 (54.6%) | 57'410 (55.1%) | 59'747 (55.7%) | 63'389 (56.2%) | 67'583 (56.7%) |

*Note*: CH = Switzerland; Intmed = rural center with urban area; Nurs = Nursing home; TCA = tricyclic antidepressants (N06AA), SSRI = selective serotonin reuptake inhibitors (N06AB), MAOI = monoamine oxidase inhibitors (N06AG), Others = Other ADs (NA06AX).
